# Trends and effect of marginalization on diabetes mellitus-related mortality in Mexico from 1990 to 2019

**DOI:** 10.1038/s41598-022-12831-z

**Published:** 2022-06-02

**Authors:** Eduardo Gutiérrez-León, Ricardo Antonio Escamilla-Santiago, Pablo Martínez-Amezcua, Usama Bilal, Mariana Lazo, Rafael Ogaz-González, Malaquías López-Cervantes

**Affiliations:** 1 Department of Public Health, School of Medicine, National Autonomous University of Mexico, Mexico City, Mexico; 2 PECEM (MD/PhD), School of Medicine, National Autonomous University of Mexico, Mexico City, Mexico; 3 Division of General Medicine, Columbia University Irving Medical Center, New York, NY USA; 4 Urban Health Collaborative, Drexel Dornsife School of Public Health, Philadelphia, PA USA; 5 Department of Epidemiology and Biostatistics, Drexel Dornsife School of Public Health, Philadelphia, PA USA; 6 Department of Community Health and Prevention, Drexel Dornsife School of Public Health, Philadelphia, PA USA; 7 Sixth Floor, Building B-Department of Public Health, School of Medicine, Copilco Universidad, 411A Circuito Escolar, Coyoacán, 04360 Mexico City, Mexico

**Keywords:** Diabetes, Risk factors, Epidemiology

## Abstract

Diabetes mellitus (DM) is currently one of the leading causes of mortality worldwide. However, the disease evolves differently across countries. This study intends to characterize the trends and assess the potential effects of marginalization on DM mortality between 1990 and 2019 in Mexico. We analyzed death certificates that listed DM as the underlying cause of death (N = 1,907,173), as well as the extent to which DM mortality changes were associated with marginalization through an age-period-cohort analysis. DM mortality increased in Mexico between 1990 and 2019; the change was faster in the first half and slowed down after 2004. The highest marginalization quintiles drove the changes in DM mortality trends during the study period, with a higher risk of dying in these quintiles as age increased. In recent cohorts, the highest marginalization quintiles doubled the risk of dying from DM as compared to the lowest. Renal complications was the main death driver among persons with DM, with a marked increase between 1999 and 2001. In conclusion, Mexico continues to have a substantially high DM mortality, but its pace slowed over time. Moreover, subnational differences in marginalization can partially explain such a trend.

## Introduction

Around 451 million adults lived with diabetes mellitus (DM) worldwide in 2017, an 8.7% increase compared to 2015^1,2^. These numbers are expected to increase by 53.7% by 2045^2^. In 2017, there were 5 million DM deaths worldwide, a third of wich occurred in people under the age of 60^2^. According to the International Diabetes Federation 2019, Mexico is one of the countries with the highest prevalence of DM, and it is expected to remain as such for the next 3 decades^3,4^. Additional concern stems from the increasing DM mortality in the country. In 2016, for example, DM ranked as the first cause of death for females and the second cause for males, particularly in the northern states^5,6^. DM also has significant economic repercussions. With ~ $17 billion USD Mexico was the sixth country with the highest total spending on health due to DM in 2019^3^.

The prevalence of DM in Mexico increased steadily over the last decades. According to the National Health and Nutrition Surveys of Mexico (ENSANUT), the prevalence of diagnosed DM has gone from 7.5 to 10.3% between 2000 and 2018^7–10^. Data on DM incidence in Mexico is scarce^11^; yet, the relationship between incidence, prevalence, and mortality, suggests that incidence is increasing concurrently. The constant and significant increase in mortality and prevalence suggests that longevity in people with DM is not enough to maintain these trends, pointing towards the involvement of a marked incidence.

Prior characterizations of national DM mortality trends in Mexico are limited to short periods, do not explore potential drivers, and do not examine sub-national variations. Mexico is a heterogeneous nation in terms of development and urbanization (marginalization)^12,13^. Thus sub-national DM mortality trends should inform policies and resource allocation, which warrants further research.

There is limited evidence for age-cohort-period analyses of DM mortality in recent years, with higher results found in residents of urban areas (except for a more favorable pattern in urban population of China)^14,15^. The age-cohort-period is a statistical tool to extract information hidden in age-adjusted DM-related mortality. The age effect indicates varying risks of diverse outcomes throughout distinct life periods; the period effect reflects population-wide exposure at a circumscribed point in time; the cohort effect generally represents the disparities of risk across birth cohorts. The marginalization degree on DM mortality makes it possible to identify whether marginalization provokes disparities in the analyzed effects^16,17^.

We examined the extent to which DM mortality rates and changes are associated with population-level characteristics through marginalization.

## Results

There were 1,907,173 deaths due to DM between 1990 to 2019. Out of that total, 24,461 (1.28%) were excluded because there was a mismatch between reported and calculated age. Most of the exclusions (82.64%) belonged to the initial years of the study period (1990–1995). Out of the 1,882,712 DM deaths registered between 1990 and 2019, 26.66% belonged to people under 60 years of age; with a mean of 67.55 years (standard deviation [SD] ± 13.55) at the age of death.

**Figure 1:**
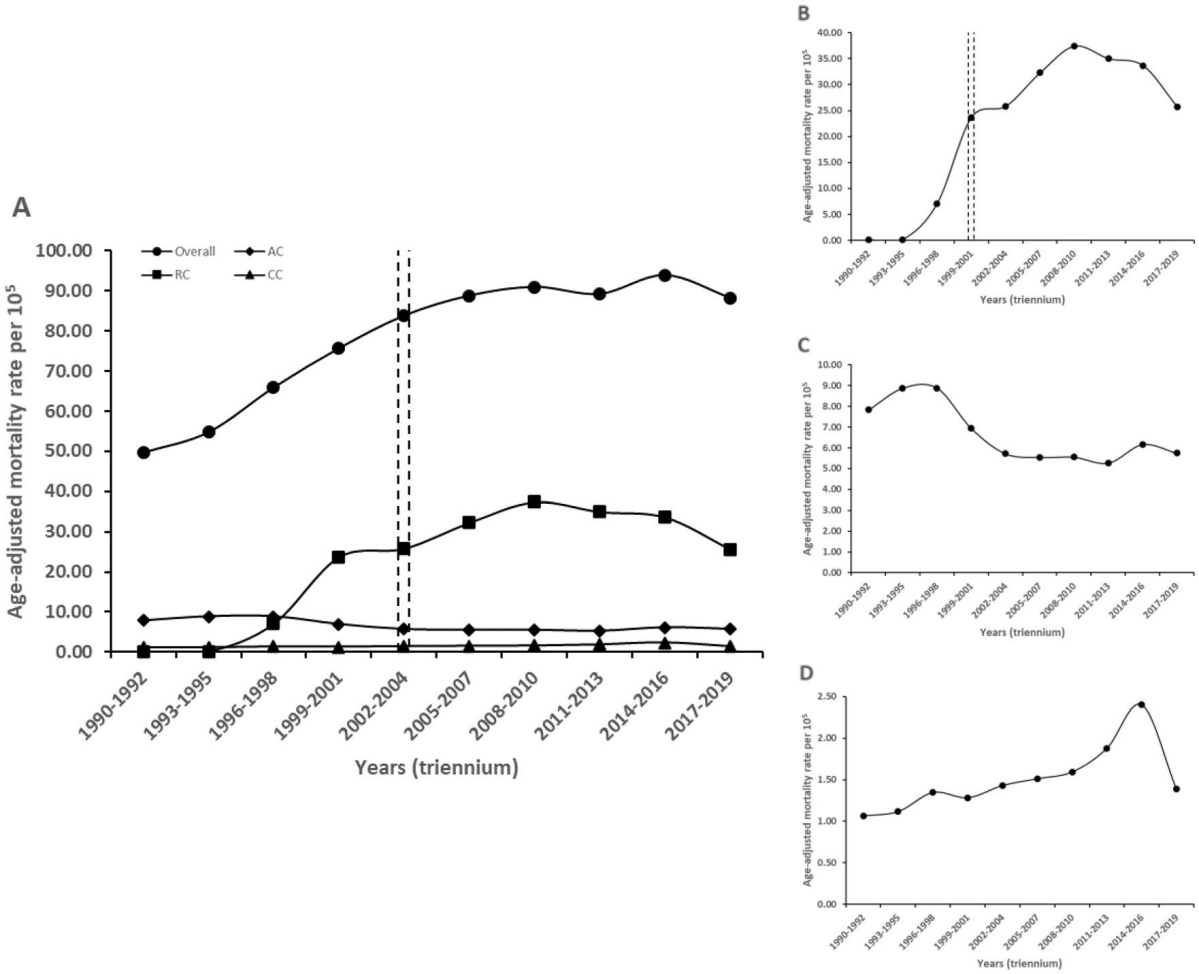
Trends in age-adjusted mortality rates for diabetes mellitus and for specific complications recorded as primary cause in Mexico, 1990–2019. This figure shows age-adjusted DM mortality rate for specific complications registered as a primary cause of death in Mexico from 1990 to 2019, with respect to the national rate (**A**). Likewise, a smaller range of the Y-axis shows the age-adjusted mortality rate for DM deaths that registered renal complications (**B**), acute complications (**C**) and circulatory complications (**D**) as a primary cause of death. Data markers represent observed rates by triennium. Lines are fitted rates by triennium based on joinpoint analysis. *DM* diabetes mellitus, *AC* acute complications, *RC* renal complications, *CC* circulatory complications. ^a^Age-adjusted mortality rates are shown per 100,000 population. ^b^The dotted lines indicates the inflection point in the trend.

Figure 1 shows that, at the national level, the age-adjusted DM mortality rate rose from 49.80 (95% confidence interval [CI] 49.42–50.17) to 88.23 per 100,000 inhabitants (CI 87.92–88.55), which is a 77.17% increase from1990 to 2019. There was an inflection point at 2002–2004. The annual percent change (APC) was steeper from 1990 to 2004 (15.5%, 95% CI 10.2–21.1%), than from 2002 to 2019 (0.9%, − 1.1 to 3.1%). Male had a higher age-adjusted DM mortality compared to female (94.8 vs 82.3/100,000). Both sexes initially experienced similar trends of increasing mortality over time, but the pace slowed down for females after 2004. In males, such deceleration was observable only after 2007. The most frequent way of dying, as shown by the death certificates, was to develop renal complications (25.7/100,000). Other complications included acute glycemic and macrovascular events (5.7 and 1.4/100,000 respectively). Only renal complications showed an inflection point after 2001, with an APC of 1.4% (95% CI − 8.4 to 12.3%). Circulatory complications had a continuous increase from 1990 to 2019 (6.6%, 0.8–12.8%), while the category of acute complications experienced a downward trend (− 4.9%, − 7.9 to − 1.8%), as presented in Table 1 and Fig. 1.

**Table 1:** Mortality rate and annual percentage change for diabetes mellitus in Mexico.

	Mortality rate		First APC		Second APC	
	1990–1992 [95% CI]	2017–2019 [95% CI]	Period^a^	APC [95% CI]	Period^a^	APC [95% CI]
National	49.80 [49.42–50.17]	88.23 [87.92–88.55]	1990–2004	15.5 [10.2–21.1]	2002–2019	0.9 [− 1.1 to 3.1]
**By complication**						
Acute	7.83 [7.68–7.98]	5.75 [5.67–5.83]	1990–2019	− 4.9 [− 7.9 to − 1.8]	–	–
Renal	0.08 [0.06–0.09]	25.71 [25.54–25.88]	1990–2001	375.1 [− 94.7 to 42,300.1]	1999–2019	1.4 [− 8.4 to 12.3]
Circulatory	1.06 [1.01–1.12]	1.39 [1.35–1.43]	1990–2019	6.6 [0.8–12.8]	–	–
**Sex**						
Men	47.16 [46.63–47.70]	94.81 [94.33–95.29]	1990–2007	14.2 [10.5–18.0]	2005–2019	1.4 [− 1.4 to 4.3]
Woman	51.81 [51.29–52.33]	82.26 [81.85–82.67]	1990–2004	14.4 [9.5–19.5]	2002–2019	− 0.8 [− 2.8 to 1.1]

Marginalization	1990–1994 [95% CI]	2015–2019 [95% CI]	Period^a^	APC [95% CI]	Period^a^	APC [95% CI]
Very low	72.06 [71.23–72.89]	83.69 [83.14–84.24]	1990–2009	10.8 [8.5–13.2]	2005–2019	− 8.7 [− 11.4 to − 6.0]
Low	56.78 [56.24–57.33]	89.64 [89.19–90.09]	1990–2004	26.1 [− 40.6 to 167.9]	2000–2019	0.1 [− 20.4 to 25.9]
Medium	40.70 [39.58–41.85]	92.67 [92.04–93.29]	1990–2019	9.6 [1.9–17.9]	–	–
High	43.60 [43.02–44.19]	97.04 [96.51–97.57]	1990–2004	37.0 [− 19.9 to 134.3]	2000–2019	7.0 [− 6.3 to 22.0]
Very high	38.61 [38.12–39.11]	91.01 [90.22–91.81]	1990–2019	16.6 [10.6–23.0]	–	–

Because it was categorized into periods, the first and second APC overlaps with the end and the beginning of the period where the inflection point was found. The start of the second APC is at the beginning of the period where the inflection point is found. The first APC ends at the end of the period where the inflection point is found.

*APC* annual percentage change, *CI* confidence interval.

^a^The two annual percentage changes mark the beginning and end of each period until the turning point.

At the national level, marginalization quintiles show that the highest age-adjusted DM mortality belonged to the very low quintile (72.1/100,000), while the lowest mortality rate corresponded to the highest quintile (38.6/100,000) between 1990 and 1994 (Table 1 and Supplementary Fig. S1). The situation then reversed and, by the end of the study period (2015–2019), the highest rate was in the high quintile (97.0/100,000) and the lowest rate was in the very low quintile (83.7/100,000). Hence, marginalization is one of the most important drivers of the DM mortality rate in Mexico, as underscored by three main observations. First, the medium and very high quintiles did not show any inflection point. Overall, the average annual percent change (AAPC) for the highest quintile (16.6%, 95% CI 10.6–23.0%) was greater than the medium quintile (9.6%, 1.9–17.9%) across the period. Second, mortality in the high and low quintiles experienced a stable ascent after 2004 (7.0%, − 6.3 to 22.0%; and 0.1%, − 20.4 to 25.9%, respectively). Third, the very low quintile was the only one with an improvement in mortality, experiencing a consistent decrease of − 8.7% (− 11.4 to − 6.0%).

**Figure 2:**
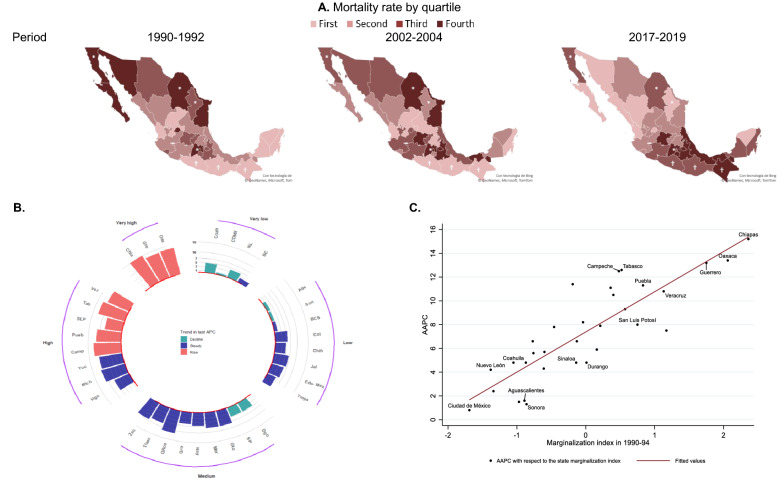
Age-adjusted mortality rates at the state level and correlation for the AAPC of diabetes mellitus mortality and marginalization in Mexico, 1990–2019. (**A**) The age-adjusted mortality rate for DM by state in Mexico categorized by quartiles during the periods 1990–1992, 2002–2004, and 2017–2019. Likewise, it shows the AAPC by state, categorized by marginalization quintiles (**B**). The scatter plot shows the state AAPC with respect to the marginalization index during the 1990–1994 period. States that showed a significant trend (*p* < 0.05) in their last APC were labeled with their name (**C**). *States that predominated in the very low marginalization quintile throughout the period; ^†^states that predominated in the very high marginalization quintile throughout the period. *DM* diabetes mellitus, *APC* annual percentage change, *AAPC* average annual percentage change.

To better outline the worsening trends in mortality from DM across the country and regarding the marginalization quintiles, we present a series of heat maps (Fig. 2) with the DM death rate at the state level over time. We also depict the AAPCs by quintile of marginalization and state. The states with the greatest AAPCs were in the very high marginalization quintile. Those are Chiapas (15.2%, 10.5–20.0%), Oaxaca (13.4%, 11.5–15.3%) and Guerrero (13.2%, 9.9–16.6%). Contrastingly, the three states with the least worsening are those in the low or very low marginalization quintiles Those are Baja California Sur (1.50%, − 1.60 to 4.80%), Sonora (1.30%, − 2.10 to 4.90%), and Mexico City (0.80%, − 0.80 to 2.50%). Finally, the increase in the AAPC experienced a very high positive correlation with the marginalization index at the state level.

On the other hand, all the components of the marginalization index had significant positive correlations (P < 0.001) with AAPCs at the beginning of the study period As shown in Supplementary Table S1, the highest values corresponded to education (population with less than basic education [0.86]), followed by household characteristics and income (having a paid job with an income no greater than two minimum wages [0.62]).

**Figure 3:**
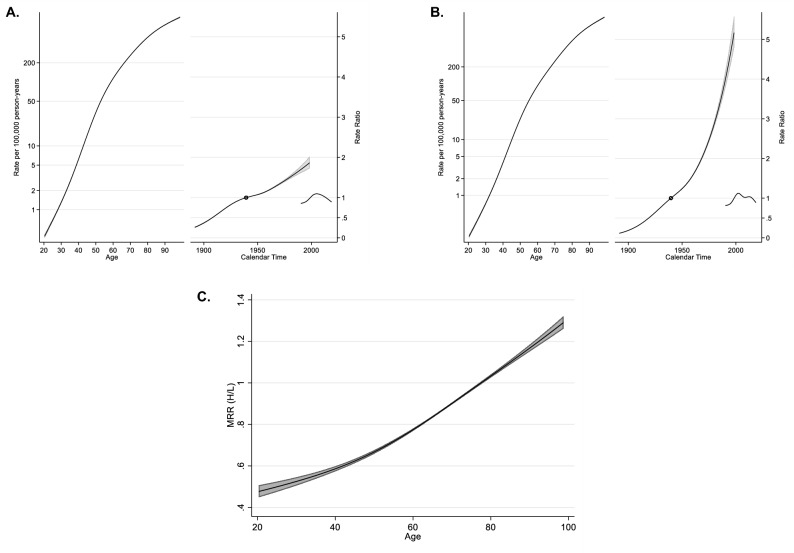
Age-period-cohort analysis for diabetes mellitus mortality rate in the very low and low quintile of marginalization in relation to medium, high and very high in Mexico, 1990–2019. (**A**) The age-period-cohort model for the population with the lowest marginalization (very low and low quintile). (**B**) The age-period-cohort model for the population with the highest marginalization (medium, high, and very high quintiles) between 1990 and 2019. The leftmost solid line is the age effect, the longest of the solid lines on the rate ratio half of the graph is the cohort effect, and the shortest line is the period effect. Finally, (**C**) shows the time-dependent mortality rate ratio for the interaction of the highest marginalization quintiles with respect to the lowest quintiles by age. The shaded gray area indicates the 95% confidence interval. *DM* diabetes mellitus, *MRR* mortality rate ratio.

For the age-period-cohort analysis, DM mortality rates were divided in quintiles according to state marginalization levels. Findings are summarized in Fig. 3, as follows. First, DM mortality rates went up with increasing age. The lowest quintiles had an estimated mortality value of 1/100,000 at younger ages but had 30-fold higher mortality values in the highest marginalization quintiles (28 vs 32 years). For the category of 60-year-old individuals, the lowest quintiles had relatively higher DM mortality than the highest quintiles (121.2 vs 99.3/100,000). Contrastingly, Supplementary Table S2 shows that, at the age of 80 years, DM mortality was very similar among the highest and lowest marginalization quintiles (617.1 vs 519.8/100,000). Second, when comparing rate ratios across marginalization quintiles, we found much greater values for the same birth cohorts over time, following the 1939 birth cohort (in which the marginalization quintiles had rate ratios equal to one). For example, Supplementary Table S3 shows that the 1950 birth cohort had a rate ratio of 1.25 (95% Confidence Interval 1.25–1.26) in highest vs 1.08 (1.07–1.09) in lowest quintiles, the 1965 cohort had a rate ratio of 1.86 (1.83–1.88) in highest vs 1.26 (1.25–1.28) in lowest quintiles, and 1980 had a ratio of 2.94, (2.86–3.02) in highest vs 1.52 (1.48–1.56) in lowest quintiles. Third, the period effect does not show wide differences in the rate ratios by marginalization quintiles. However, mortality in the highest quintiles showed a major difference after 1997, which persisted until 2003 over the lowest quintiles. In 2002, there was an inflection point at the national level, and the period effect is the greatest for the highest quintiles as compared to the lowest quintiles. Supplementary Table S4 shows that it is 1.12 (1.11–1.13) vs 1.08 (1.07–1.09).

Finally, time-dependent mortality rates for the interaction among the highest and the lowest quintiles of marginalization showed greater rates for the highest quintiles until the age of 78 years, reaching a DM mortality rate ratio of 1.29 (95% CI 1.26–1.32) at the age of 98 years (Fig. 3). However, the highest quintiles of marginalizaition had about 11% lower DM mortality than the lowest quintiles across the 1990–2019 period in Mexico, when adjusting for age, birth cohort end period.

## Discussion

DM mortality has increased dramatically in Mexico over the last 30 years. Such trends, however, are temporally and geographically heterogeneous. While mortality increased between 1990 and 2019, the change was faster during the first decades and slowed after 2004. Renal complications were the main cause of death reported in certificates. Moreover, both the rate of change and the temporal patterns themselves differ widely by geography. We found a discrepancy in the age-period-cohort analysis by marginalization quintiles, with the most unfavorable outcomes for the people in the highest quintiles, as well as a correlation between the marginalization index and the trends that partly explain the change in geography and the high DM mortality.

At the international level, countries like the United States, Brazil, and Spain, have reported a decrease in their DM mortality rates. Such decreases have been attributed to improvements in care systems and to greater control of the disease and its different risk factors^18–20^. In contrast, Mexico has underwhelming levels of DM control. For example, only 22% of patients have glycated hemoglobin [HbA1c] < 7%)^21,22^, suggesting no improvement in DM management. Previous studies found that Mexicans with diabetes had higher HbA1c levels compared to other countries (9% in Mexico vs. 7.08% in the United Kingdom)^23,24^. Moreover, Mexican surveys using HbA1c have found little differences in DM control across states (e.g. Tabasco and Mexico City, with 22.73 and 24.00% respectively, Supplementary Table S4)^22^. On the other hand, data using longitudinal assessments of HbA1c in Mexico City shows that DM control has improved over time, going from an average HbA1c of 8.3 in 1998–2004 to 7.2% in 2015–2019^25^. These results extrapolate to other states in the very low and low marginalization quintiles given shared characteristics like greater access to medical services, which implies a DM mortality decrease.

Our findings regarding monotonic increases in DM mortality in eight states are consistent with the increasing prevalence of diabetes (e.g., Tabasco, a state with a steady increase in DM mortality had a DM prevalence of 6.2%, 9.4%, and 12.1%, in 2006, 2012, and 2018, respectively)^8–10^. A potential explanation is that the states with increased mortality and prevalence are in the highest marginalization quintiles, with limited access to health systems and preventive education. On the other hand, states with more stable mortality rates and prevalence (e.g., Mexico City: 8.9%, 12.3%, and 12.7%, for 2006, 2012, and 2018, respectively)^8–10^ are in the lowest marginalization quintiles. Thus, the discrepancy between trends in mortality and prevalence can be partially explained by varying demographic conditions (e.g. education, incomes). Other potential explanations include differences in the prevalence of DM risk factors, leading to different incidences. However, there are almost no studies to support these hypotheses.

The age-period-cohort analysis suggests that the recent birth cohorts are at greater risk of dying from DM, as compared with the reference cohort. In this context, the highest marginalization quintiles have a twofold greater risk of death than the lowest quintiles. Likewise, the aging course has a significant effect on DM mortality regardless of the marginalization quintile. This can be explained by the increase in life expectancy, as a higher risk of developing DM and dying is associated with aging. The effect on birth cohorts has to do with the increasing frequency of other conditions, which are also considered risk factors for the development of DM and death (e.g. obesity)^26^.

Finally, it is important to mention that the main cause of death in people with DM in Mexico is renal complications, coinciding with reports from the Mexican cohort study^24^. Contrastingly, other countries report cardiovascular complications as the main cause of death^27^. Given the trends observed in death certificates during this study, it is necessary to conduct further research in Mexico to explicate the relationship between renal insufficiency and DM, as well as to explore the importance of the latter in regard to the incidence of heart disease.

This is the first study showing evidence of a decrease in DM mortality in Mexico in recent years. However, it is worth noting the eight states with a clear increase in their trend up to 2019. This finding has great implications for the health system. Decision-makers in these states should consider screening at earlier ages and strengthening institutional programs focused on DM management. We emphasize that states that decreased mortality might only reflect demographic measures. Other indicators should be identified when evaluating state DM control programs.

The main strength of this study is the richness of the data, which accounted for over 1.8 million DM deaths over 30 years, making it possible to evaluate other variables of interest like age and marginalization. The reliability shown by death certificates in Mexico stands out. When performing a sub-analysis in the same period, we considered only 1.71% of 15,527,101 deaths due to unclassified conditions (chapter R of the International Classification of Disease [ICD]). Moreover, Mexico has a high-quality mortality coverage^28^. The above implies that, in general, it is possible to determine a cause of death and cover the majority of DM deaths.

Our study also has limitations. First, there is the potential misclassification of the underlying cause of death. Even though trained personnel fill death certificates, it is still likely for the cause of death to be improperly reported. For example, we observed a drastic decrease in DM mortality in 2018–2019. This sharp decrease was not coherent with the trends we observed in the previous years. However, the decrease was consistent across all states. To smooth these potential year-to-year variations, we grouped deaths by triennia. Another reason behind the decline in DM deaths is a large number of violent deaths in Mexico (36,685 occurred in 2018 alone)^29^, which may act as a competing risk. Second, despite considering that the marginalization shows an effect on the obtained time trend, it must be emphasized that this is based on statewide parameters, which may not be entirely accurate for all the population in each state. Third, this is an ecological study, which does not allow to make inferences at the individual level. However, our key objective was to assess contextual trends at the national and state level.

In conclusion, Mexico continues to be a country with a very high DM mortality, and it could even be classified among the highest in the world (Supplementary Fig. S2)^30^. Given current trends, it seems plausible that DM mortality will decline in the following years, as hypothesized through the current stagnation and the decline in some states. However, a number of states still show continued increases with renal complications as the leading cause of death. This represents an opportunity for population-level diabetes prevention strategies. We observed that marginalization drove these trends, but we could not rule out other aspects, including changes in diabetes control or incidence, which would warrant future studies.

## Methods

### Study design and setting

This is an ecological epidemiological study of the temporal trends of DM mortality rates in Mexico from 1990 to 2019. The data were obtained from the vital registration system of the National Institute of Statistics, Geography, and Informatics (INEGI).

### Outcomes

The main outcome was all registered deaths in which DM, or its complications, were listed as the underlying cause of death. From 1990 to 1998, Mexico used the ninth revision of the ICD. During this period, code 250 was used to identify DM deaths. The following extended codes were used for specific complications: 2501–2502 (acute), 2504 (renal), and 2507 (circulatory). Between 1999 and 2019, Mexico used the ICD-10, and the following codes were used: E10–E14. The following number was added to the previous codes for specific complications: 0 and 1 (acute), 2 (renal), and 5 (circulatory)^31,32^. The cause of death is determined at the time of death by trained personnel, which includes physicians and government-licensed personnel.

As a quality control strategy, we excluded deaths certificates if the age reported and the age calculated from their date of birth did not match (acceptable margin of ± 1 year of difference).

### Marginalization index

We used a set of population measures representing the level of marginalization to investigate the extent to which changes in demographic and economic characteristics were related to DM mortality rates and trends.

The marginalization index at the state level was calculated in 1990, 1995, 2000, 2005, 2010, and 2015 by the Mexican National Population Council (CONAPO), obtaining quintiles of marginalization categorized as very low, low, medium, high, and very high (states with fewer resources). The marginalization index is calculated based on the level of access to resources in four domains: education (% illiterate, %without primary education), housing (% of people living in houses without proper sewage system, electricity, potable water, overcrowding, dirt floor), population size (< 5000 residents), and income (% of employed residents with a daily income < $140.20 MX pesos [~ $9.63 USD]). In 2015, 10 of 32 Mexican states were in the very high and high quintiles, while 4 were in the very low marginalization quintile^33^.

We merged INEGI’s vital registry datasets with CONAPO’s marginalization datasets using a common and unique identifier assigned to each state. The level of marginalization was assigned to each death according to the last state of residence. All deaths were linked correctly.

### Statistical methods

We conducted this analysis in four steps. First, we computed crude and age-adjusted mortality rates due to the specific and total causes of DM, with the respective 95% CI over 3-year periods from 1990 to 2019. We did it for the whole country and by state using formulas developed by Tiwari, Clegg, and Zou^34^. As denominators, we used population projections and intercensal estimations of the total population by age, sex, and state from 1970 to 2050, published by CONAPO^35^. For the age-adjusted mortality rates, we used the 2000–2025 WHO world standard population^36^.

Second, we pooled state trends into 3-year periods to characterize changes in the age-adjusted mortality trends due to the specific and total causes of DM for the 32 states of Mexico. We computed APC, AAPC, and their 95% CI using the 4.7.0.0 version of the Joinpoint Regression Program^37^. We considered significant findings in the joinpoint at *P* < 0.05.

Third, we compared the national and state level crude and age-adjusted DM mortality rate by marginalization quintiles. The mortality rate by marginalization quintiles was estimated by 5-year periods for each state (e.g., 1990–1994) to match the calculation of marginalization by the CONAPO. As in the previous step, we estimated the APC and AAPC for the mortality trend by state and marginalization quintiles. Likewise, we evaluated the correlation between AAPC and marginalization index and with each of its components by state at the national level.

Fourth, we performed an age-period-cohort analysis for the marginalization-stratified by lowest (low and very low quintiles) and highest (medium, high, and very high quintiles). The general age-period-cohort model can be described using the following equation: ln {*λ*(a, p)} = *f*(a) + *g*(p) + *h*(c). This analysis was performed using the method proposed by Carstensen, with the advantage of analyzing the effects as continuous variables to avoid the overparameterization and, consequently, to exclude one of the terms with the use of the Lexis diagram and restricted cubic splines to model the three variables, which facilitates interpretation, estimation, and forecasting^38^. The evaluated outcomes were mortality rates, rate ratios, and mortality rate ratios due to DM, with their respective 95% CI.

The reference period was 1990, and the reference birth cohort was that born in 1890. The first year of the study period was 1990, because that is the first year for which complete data on registered deaths is available as an individual data base. A new death certificate was introduced in 1987 and then the procedures to capture deaths and integrate a complete data base were implemented and took up to 1990, to have the first year with complete data. Thereafter, from 1990 to 2019 we considered that we could have a reasonable time window to carry out a study of the kind we are presenting. The selection of the birth cohort 1890 was taken upon the assumption that almost any subject then born would be dead by the time of the beginning of the study, giving us the possibility to have unique combinations of age and period.

We conducted analyses in the 15.1 version of Stata (StataCorp LLC, USA), as well as in the 4.7.0.0 version of the Joinpoint Regression Program (https://surveillance.cancer.gov/joinpoint/). Joinpoint analysis determines the inflection points in the trends, from the APC or slope, between three or more measurements using piecewise linear regression. We stipulated a maximum of one joinpoint because the improvement was not statistically significant when adding an extra one. Thus, each of the joinpoints and their corresponding changes in the trend could be interpreted as significant. The sequential permutation test procedure to choose the best joinpoint follows the following equation: *E*[*y*[*x*] = *β*_0_ + *β*_1_*x* + *δ*_1_(*x* − *Τ*_1_)^+^  + ··· + *δ*_*κ*_(*x* − *Τ*_1*κ*_)^+^. We used the 15.1 version of Stata, Microsoft Excel, and the 3.6.3 version of R to create figures.

The protocol was approved by the Ethics and Research Committees of the Faculty of Medicine at the National Autonomous University of Mexico with project number: FM/DI/031/2020. We did not request written informed consent or patient consent because we worked with open mortality databases provided by the Mexican Government, which censor personal information.

The present study followed the items proposed in the Strengthening the Reporting of Observational Studies in Epidemiology (STROBE), and the Reporting of studies Conducted using Observational Routinely-collected health Data (RECORD) Statements, guidelines for reporting observational studies.

### Research ethics approval

The protocol was approved by the Ethics and Research Committees of the Faculty of Medicine at the National Autonomous University of Mexico with project number: FM/DI/031/2020. We did not request written informed consent or patient consent because we worked with open mortality databases provided by the Mexican Government, which censor personal information.

### Relevant guidelines

The present study followed the items proposed in the Strengthening the Reporting of Observational Studies in Epidemiology (STROBE) and the Reporting of studies Conducted using Observational Routinely-collected health Data (RECORD) Statements, guidelines for reporting observational studies.

## Supplementary Information

Supplementary Information.

## Data Availability

Data are available in a public, open access repository. All data relevant to the study are included in the article or uploaded as online supplemental information.
