# Supplementary material for: Trends and effect of marginalization on diabetes mellitus-related mortality in Mexico from 1990 to 2019
Source: Sci Rep. 2022 Jun 2;12:9190. doi: 10.1038/s41598-022-12831-z (PMC9163326; doi:10.1038/s41598-022-12831-z)
Supplement: Supplementary file 1 — Supplementary Information. [file 41598_2022_12831_MOESM1_ESM.pdf]

Article title: Trends and effect of marginalization on Diabetes Mellitus-related Mortality in Mexico from 1990 to 2019: A Joinpoint and Age-Period-Cohort Analysis.

Author information: Eduardo Gutiérrez-León MD[1,2]; Ricardo Antonio Escamilla-Santiago MD, PhD[1]; Pablo Martínez-Amezcua MD, PhD[3]; Usama Bilal MD, PhD[4,5]; Mariana Lazo MD, PhD[4,6]; Rafael Ogaz-González MSc[1]; Malaquías López-Cervantes MD, PhD[1]*.

1. Department of Public Health, School of Medicine, National Autonomous University of Mexico, Mexico City, Mexico.
2. PECEM (MD/PhD), School of Medicine, National Autonomous University of Mexico, Mexico City, Mexico.
3. Division of General Medicine, Columbia University Irving Medical Center, New York, NY, USA.
4. Urban Health Collaborative, Drexel Dornsife School of Public Health, Philadelphia, Pennsylvania, United States.
5. Department of Epidemiology and Biostatistics, Drexel Dornsife School of Public Health, Philadelphia, Pennsylvania, United States.
6. Department of Community Health and Prevention, Drexel Dornsife School of Public Health, Philadelphia, Pennsylvania, United States.

***Corresponding author:** Malaquías López-Cervantes

Supplementary material

Figure S1. Crude and age-adjusted mortality rates for diabetes mellitus in Mexico by marginalization quintiles from 1990-2019.

Table S1. Pearson correlation coefficients for the marginalization index and its components from 1990 to 1994 and 2005 to 2009 with respect to the average annual percentage change in mortality due to diabetes mellitus from 1990-2019 in Mexico.

Table S2. Effect of age expressed as mortality rates by quintiles of lowest and highest marginalization for diabetes mellitus in Mexico, 1990-2019.

Table S3. Effect of birth cohort expressed in rate ratios by lowest and highest marginalization quintiles for diabetes mellitus mortality in Mexico, 1990-2019.

Table S4. Effect of period expressed in rate ratios by quintiles of lowest and highest marginalization for diabetes mellitus mortality in Mexico, 1990-2019.

Table S5. Proportion of individuals corresponding to different levels of HbA1c and state entity according to ENSANUT-2012.

Figure S2. Age-adjusted mortality rates for diabetes mellitus in Mexico and selected countries from 1990 to 2016. Provided by WHO.

Figure S1

A. Crude mortality rate **B. Age-adjusted mortality rate**

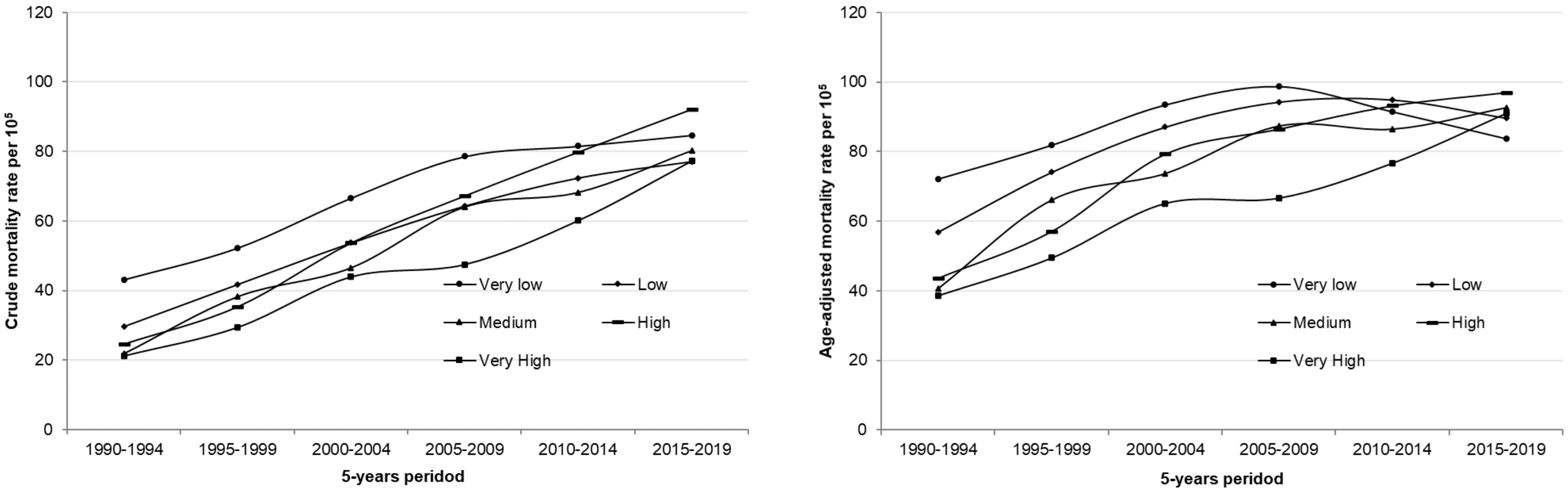

Data markers represent observed rates by quinquennium for each one of the deprivation quintiles.

[a] Crude and age-adjusted mortality rates are shown per 100,000 population.

Table S1

Pearson correlation coefficient	Average annual percentage change	*P* value	Average annual percentage change	*P* value
	Period 1990-94		Period 2005-09	
Marginalization index in 1990-94.	0.86	<0.001	0.85	<0.001
Percentage of population illiterate.	0.85	<0.001	0.84	<0.001
Percentage of population without basic education.	0.86	<0.001	0.84	<0.001
Percentage of population without sewage and sanitation service.	0.72	<0.001	0.57	<0.001
Percentage of population without electricity.	0.64	<0.001	0.57	<0.001
Percentage of population without piped water.	0.79	<0.001	0.70	<0.001
Percentage of population with some level of overcrowding.	0.86	<0.001	0.85	<0.001
Percentage of population living in dwellings with dirt floors.	0.75	<0.001	0.68	<0.001
Percentage of population in towns with less than 5,000 inhabitants.	0.72	<0.001	0.72	<0.001
Percentage of employed population with income up to two minimum wages.	0.62	<0.001	0.81	<0.001

Table S2

	Lowest marginalization quintiles*			Highest marginalization quintiles**		
Age	DM mortality / 100,000 inhabitants	95% LCI	95% UCI	DM mortality / 100,000 inhabitants	95% LCI	95% UCI
**20**	0.40	0.38	0.42	0.19	0.18	0.20
**21**	0.45	0.43	0.48	0.22	0.21	0.23
**22**	0.51	0.49	0.54	0.25	0.24	0.27
**23**	0.58	0.55	0.60	0.29	0.28	0.30
**24**	0.65	0.63	0.68	0.33	0.32	0.35
**25**	0.74	0.71	0.77	0.38	0.37	0.40
**26**	0.84	0.81	0.87	0.44	0.43	0.46
**27**	0.96	0.93	0.99	0.51	0.49	0.53
**28**	1.09	1.06	1.12	0.59	0.57	0.61
**29**	1.25	1.22	1.28	0.68	0.67	0.70
**30**	1.43	1.40	1.47	0.80	0.78	0.82
**31**	1.65	1.61	1.69	0.93	0.91	0.95
**32**	1.90	1.86	1.94	1.09	1.06	1.11
**33**	2.20	2.16	2.25	1.28	1.25	1.30
**34**	2.57	2.52	2.62	1.50	1.47	1.54
**35**	3.00	2.94	3.06	1.78	1.75	1.82
**36**	3.52	3.46	3.59	2.12	2.08	2.16
**37**	4.15	4.08	4.23	2.53	2.48	2.58
**38**	4.91	4.83	5.00	3.03	2.98	3.09
**39**	5.83	5.74	5.92	3.64	3.58	3.71
**40**	6.93	6.82	7.04	4.38	4.31	4.46
**41**	8.24	8.13	8.36	5.28	5.20	5.36
**42**	9.81	9.68	9.95	6.36	6.27	6.46
**43**	11.68	11.53	11.83	7.67	7.56	7.77
**44**	13.88	13.71	14.05	9.22	9.11	9.35
**45**	16.47	16.29	16.66	11.08	10.95	11.22
**46**	19.50	19.29	19.71	13.28	13.13	13.43
**47**	23.01	22.78	23.25	15.86	15.69	16.04
**48**	27.05	26.78	27.31	18.88	18.68	19.08
**49**	31.64	31.35	31.94	22.36	22.14	22.59
**50**	36.82	36.49	37.15	26.35	26.09	26.61
**51**	42.59	42.22	42.96	30.86	30.57	31.16
**52**	48.93	48.52	49.34	35.92	35.59	36.25
**53**	55.86	55.41	56.31	41.54	41.18	41.90
**54**	63.37	62.89	63.86	47.75	47.35	48.15
**55**	71.49	70.97	72.00	54.58	54.16	55.01
**56**	80.20	79.66	80.75	62.08	61.62	62.54
**57**	89.52	88.95	90.09	70.26	69.77	70.75
**58**	99.45	98.86	100.04	79.17	78.66	79.68
**59**	110.00	109.39	110.61	88.84	88.31	89.38
**60**	121.17	120.55	121.80	99.33	98.77	99.89
**61**	132.99	132.34	133.64	110.69	110.11	111.28
**62**	145.47	144.79	146.14	122.99	122.37	123.60
**63**	158.64	157.93	159.35	136.30	135.64	136.95
**64**	172.54	171.78	173.29	150.71	150.01	151.42
**65**	187.22	186.41	188.02	166.35	165.60	167.11
**66**	202.75	201.89	203.61	183.35	182.53	184.17

Table S2 (Continued)

	Lowest marginalization quintiles*			Highest marginalization quintiles**		
Age	DM mortality / 100,000 inhabitants	95% LCI	95% UCI	DM mortality / 100,000 inhabitants	95% LCI	95% UCI
**67**	219.20	218.29	220.12	201.86	200.98	202.74
**68**	236.66	235.69	237.63	222.04	221.10	222.99
**69**	255.12	254.10	256.15	243.99	242.98	245.00
**70**	274.60	273.52	275.70	267.79	266.71	268.88
**71**	295.10	293.94	296.27	293.54	292.37	294.71
**72**	316.60	315.34	317.87	321.28	320.00	322.57
**73**	339.09	337.70	340.48	351.09	349.66	352.53
**74**	362.52	360.97	364.08	382.99	381.36	384.64
**75**	386.87	385.11	388.63	417.01	415.12	418.90
**76**	412.07	410.07	414.08	453.12	450.92	455.32
**77**	438.05	435.77	440.35	491.28	488.72	493.85
**78**	464.74	462.16	467.34	531.42	528.45	534.40
**79**	492.04	489.12	494.97	573.42	570.01	576.86
**80**	519.83	516.57	523.10	617.13	613.23	621.05
**81**	547.99	544.39	551.61	662.34	657.94	666.77
**82**	576.38	572.43	580.35	708.79	703.87	713.75
**83**	604.84	600.54	609.17	756.20	750.75	761.70
**84**	633.26	628.61	637.95	804.32	798.30	810.38
**85**	661.60	656.55	666.68	853.04	846.41	859.73
**86**	689.83	684.34	695.37	902.31	894.96	909.72
**87**	717.96	711.93	724.04	952.08	943.88	960.35
**88**	745.97	739.29	752.70	1002.33	993.11	1011.63
**89**	773.89	766.43	781.42	1053.06	1042.62	1063.61
**90**	801.75	793.36	810.23	1104.33	1092.42	1116.37
**91**	829.60	820.11	839.19	1156.19	1142.55	1170.00
**92**	857.49	846.76	868.37	1208.76	1193.12	1224.60
**93**	885.52	873.36	897.84	1262.15	1244.24	1280.32
**94**	913.76	900.02	927.71	1316.56	1296.09	1337.35
**95**	942.32	926.84	958.05	1372.18	1348.87	1395.89
**96**	971.33	953.97	989.01	1429.28	1402.85	1456.21
**97**	1000.93	981.53	1020.71	1488.14	1458.30	1518.59
**98**	1031.27	1009.69	1053.31	1549.11	1515.57	1583.38

Abbreviations: DM, diabetes mellitus; LCI, low confidence interval; HCI, high confidence interval.

*Lowest marginalization quintiles: very low and low quintile.

**Highest marginalization quintiles: medium, high, and very high quintiles.

Table S3

	Lowest marginalization quintiles*			Highest marginalization quintiles**		
**Birth cohort**	**Rate ratio**	**LCI**	**UCI**	**Rate ratio**	**LCI**	**UCI**
**1920**	0.75	0.74	0.75	0.54	0.53	0.54
**1921**	0.76	0.76	0.77	0.56	0.56	0.57
**1922**	0.78	0.78	0.79	0.58	0.58	0.59
**1923**	0.80	0.79	0.81	0.61	0.60	0.61
**1924**	0.82	0.81	0.82	0.63	0.63	0.64
**1925**	0.83	0.83	0.84	0.66	0.65	0.66
**1926**	0.85	0.85	0.86	0.68	0.68	0.69
**1927**	0.87	0.86	0.87	0.71	0.70	0.71
**1928**	0.88	0.88	0.88	0.73	0.73	0.74
**1929**	0.89	0.89	0.90	0.76	0.75	0.76
**1930**	0.91	0.90	0.91	0.78	0.78	0.79
**1931**	0.92	0.92	0.92	0.81	0.80	0.81
**1932**	0.93	0.93	0.94	0.83	0.83	0.83
**1933**	0.95	0.94	0.95	0.86	0.85	0.86
**1934**	0.96	0.95	0.96	0.88	0.88	0.88
**1935**	0.97	0.97	0.97	0.91	0.90	0.91
**1936**	0.98	0.98	0.98	0.93	0.93	0.93
**1937**	0.99	0.99	0.99	0.95	0.95	0.95
**1938**	0.99	0.99	0.99	0.98	0.98	0.98
**1939**	1.00	1.00	1.00	1.00	1.00	1.00
**1940**	1.01	1.01	1.01	1.02	1.02	1.02
**1941**	1.02	1.02	1.02	1.04	1.04	1.05
**1942**	1.02	1.02	1.03	1.07	1.07	1.07
**1943**	1.03	1.03	1.03	1.09	1.09	1.09
**1944**	1.04	1.03	1.04	1.11	1.11	1.12
**1945**	1.04	1.04	1.05	1.13	1.13	1.14
**1946**	1.05	1.05	1.06	1.16	1.15	1.16
**1947**	1.06	1.05	1.06	1.18	1.17	1.19
**1948**	1.06	1.06	1.07	1.20	1.20	1.21
**1949**	1.07	1.07	1.08	1.23	1.22	1.24
**1950**	1.08	1.07	1.09	1.26	1.25	1.26
**1951**	1.09	1.08	1.09	1.28	1.27	1.29
**1952**	1.10	1.09	1.10	1.31	1.30	1.32
**1953**	1.11	1.10	1.11	1.34	1.33	1.35
**1954**	1.12	1.11	1.12	1.37	1.36	1.39
**1955**	1.13	1.12	1.14	1.41	1.40	1.42
**1956**	1.14	1.13	1.15	1.45	1.43	1.46
**1957**	1.15	1.14	1.16	1.48	1.47	1.50
**1958**	1.16	1.15	1.17	1.52	1.51	1.54
**1959**	1.18	1.16	1.19	1.56	1.55	1.58
**1960**	1.19	1.18	1.20	1.61	1.59	1.62
**1961**	1.20	1.19	1.22	1.65	1.64	1.67
**1962**	1.22	1.20	1.23	1.70	1.68	1.72
**1963**	1.23	1.22	1.25	1.75	1.73	1.77
**1964**	1.25	1.23	1.26	1.80	1.78	1.82
**1965**	1.26	1.25	1.28	1.86	1.83	1.88
**1966**	1.28	1.26	1.30	1.91	1.89	1.94
**1967**	1.30	1.28	1.31	1.97	1.94	2.00

Table S3 (Continued)

	Lowest marginalization quintiles*			Highest marginalization quintiles**		
**Birth cohort**	**Rate ratio**	**LCI**	**UCI**	**Rate ratio**	**LCI**	**UCI**
**1968**	1.31	1.29	1.33	2.03	2.00	2.06
**1969**	1.33	1.31	1.35	2.09	2.06	2.12
**1970**	1.34	1.32	1.37	2.16	2.12	2.19
**1971**	1.36	1.34	1.38	2.22	2.19	2.26
**1972**	1.38	1.36	1.40	2.29	2.25	2.33
**1973**	1.40	1.37	1.42	2.36	2.32	2.41
**1974**	1.41	1.39	1.44	2.44	2.39	2.48
**1975**	1.43	1.40	1.46	2.52	2.47	2.56
**1976**	1.45	1.42	1.48	2.59	2.54	2.65
**1977**	1.46	1.43	1.50	2.68	2.62	2.74
**1978**	1.48	1.45	1.52	2.76	2.70	2.83
**1979**	1.50	1.46	1.54	2.85	2.78	2.92
**1980**	1.52	1.48	1.56	2.94	2.86	3.02
**1981**	1.54	1.49	1.58	3.03	2.95	3.12
**1982**	1.55	1.51	1.60	3.13	3.04	3.23
**1983**	1.57	1.52	1.63	3.23	3.13	3.34
**1984**	1.59	1.54	1.65	3.34	3.22	3.46
**1985**	1.61	1.55	1.67	3.44	3.32	3.58
**1986**	1.63	1.57	1.70	3.56	3.42	3.70
**1987**	1.65	1.58	1.72	3.67	3.52	3.83
**1988**	1.67	1.59	1.75	3.79	3.62	3.97
**1989**	1.69	1.61	1.77	3.91	3.73	4.11

Abbreviations: LCI, low confidence interval; HCI, high confidence interval.

*Lowest marginalization quintiles: very low and low quintile.

**Highest marginalization quintiles: medium, high, and very high quintiles.

Table S4

	Lowest marginalization quintiles*			Highest marginalization quintiles**		
**Period**	**Rate ratio**	**LCI**	**UCI**	**Rate ratio**	**LCI**	**UCI**
**1990**	0.86	0.85	0.87	0.82	0.81	0.83
**1991**	0.86	0.86	0.87	0.82	0.81	0.83
**1992**	0.87	0.87	0.88	0.83	0.82	0.83
**1993**	0.88	0.88	0.89	0.84	0.84	0.84
**1994**	0.90	0.89	0.90	0.86	0.85	0.86
**1995**	0.91	0.91	0.92	0.88	0.88	0.89
**1996**	0.94	0.93	0.94	0.92	0.91	0.93
**1997**	0.96	0.96	0.97	0.96	0.96	0.97
**1998**	0.99	0.99	0.99	1.01	1.01	1.02
**1999**	1.02	1.01	1.02	1.06	1.05	1.06
**2000**	1.04	1.04	1.05	1.10	1.09	1.10
**2001**	1.07	1.06	1.07	1.12	1.11	1.12
**2002**	1.08	1.08	1.09	1.12	1.12	1.13
**2003**	1.09	1.09	1.10	1.11	1.10	1.11
**2004**	1.09	1.09	1.10	1.08	1.08	1.09
**2005**	1.09	1.09	1.10	1.06	1.05	1.06
**2006**	1.09	1.08	1.09	1.04	1.03	1.04
**2007**	1.08	1.08	1.09	1.02	1.02	1.03
**2008**	1.07	1.07	1.08	1.02	1.02	1.02
**2009**	1.06	1.06	1.06	1.02	1.02	1.03
**2010**	1.05	1.04	1.05	1.03	1.03	1.03
**2011**	1.03	1.03	1.03	1.04	1.03	1.04
**2012**	1.01	1.01	1.02	1.04	1.03	1.04
**2013**	1.00	0.99	1.00	1.03	1.03	1.03
**2014**	0.98	0.97	0.98	1.01	1.01	1.01
**2015**	0.96	0.95	0.96	0.99	0.98	0.99
**2016**	0.94	0.93	0.94	0.96	0.95	0.96
**2017**	0.91	0.91	0.92	0.92	0.92	0.92
**2018**	0.89	0.89	0.90	0.89	0.88	0.89

Abbreviations: LCI, low confidence interval; HCI, high confidence interval.

*Lowest marginalization quintiles: very low and low quintile.

**Highest marginalization quintiles: medium, high, and very high quintiles.

Table S5

Federal entity	HbA1c levels		
	< 7%	7 a 8.99%	≥ 9%
**National**	21.84%	27.30%	50.87%
**Aguascalientes**	16.67%	22.22%	61.11%
**Baja California Norte**	36.36%	22.73%	40.91%
**Baja California Sur**	25.00%	41.67%	33.33%
**Campeche**	12.50%	33.33%	54.17%
**Coahuila**	27.27%	31.82%	40.91%
**Colima**	25.64%	17.95%	56.41%
**Chiapas**	22.22%	22.22%	55.56%
**Chihuahua**	33.33%	33.33%	33.33%
**Ciudad de México**	24.00%	16.00%	60.00%
**Durango**	28.57%	28.57%	42.86%
**Guanajuato**	20.00%	46.67%	33.33%
**Guerrero**	24.00%	24.00%	52.00%
**Hidalgo**	16.67%	33.33%	50.00%
**Jalisco**	13.04%	34.78%	52.17%
**Estado de México**	16.67%	33.33%	50.00%
**Michoacán**	17.86%	21.43%	60.71%
**Morelos**	25.00%	18.75%	56.25%
**Nayarit**	7.14%	50.00%	42.86%
**Nuevo León**	26.67%	30.00%	43.33%
**Oaxaca**	11.11%	44.44%	44.44%
**Puebla**	22.73%	22.73%	54.55%
**Querétaro**	33.33%	26.67%	40.00%
**Quintana Roo**	7.14%	28.57%	64.29%
**San Luis Potosí**	28.13%	28.13%	43.75%
**Sinaloa**	28.57%	50.00%	21.43%
**Sonora**	18.75%	18.75%	62.50%
**Tabasco**	22.73%	13.64%	63.64%
**Tamaulipas**	30.77%	30.77%	38.46%
**Tlaxcala**	10.00%	20.00%	70.00%
**Veracruz**	21.05%	15.79%	63.16%
**Yucatán**	20.00%	20.00%	60.00%
**Zacatecas**	18.18%	31.82%	50.00%

Abbreviations: HbA1c, Glycated hemoglobin; ENSANUT, National Health and Nutrition Surveys of Mexico.

Figure S2

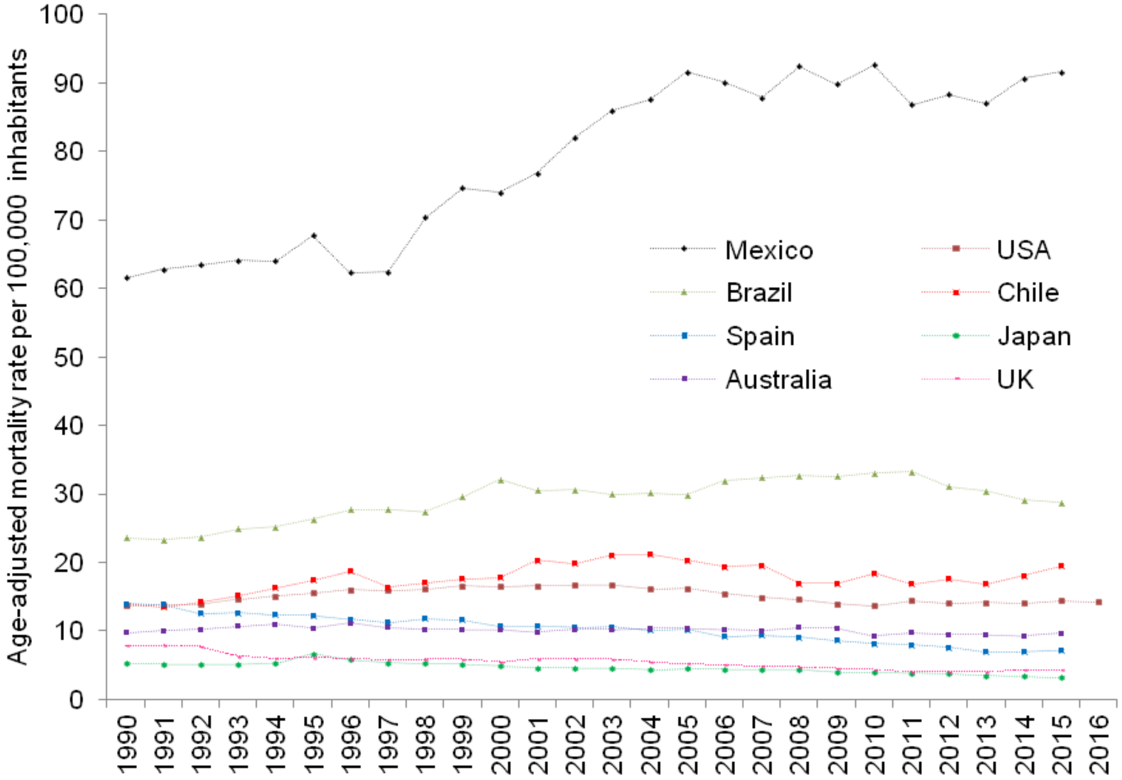

Abbreviations: USA, United States of America; UK, United Kingdom; WHO, World Health Organization.

[a] Age-adjusted mortality rates are shown per 100,000 population.

[b] The age-adjusted mortality rates for diabetes mellitus for Mexico and the other countries were obtained from the WHO mortality database.
